# Implications and pathophysiology of neuroinflammation in pediatric patients with traumatic brain injury: an updated review

**DOI:** 10.3389/fnins.2025.1587222

**Published:** 2025-04-15

**Authors:** Shalin S. Shah, Arya J. Shetty, David T. Johnston, Caroline L. Hanan, Brendan T. O’Reilly, Max A. Skibber, Ahmed T. Massoud, Banghe Zhu, Eva M. Sevick-Muraca, Jenifer Juranek, Charles S. Cox, Manish N. Shah

**Affiliations:** ^1^Department of Neurosurgery, UTHealth Houston, McGovern Medical School, Houston, TX, United States; ^2^Department of Pediatric Surgery, McGovern Medical School at UTHealth, Houston, TX, United States; ^3^Center for Molecular Imaging, The Brown Foundation Institute of Molecular Medicine, UTHealth, Houston, TX, United States

**Keywords:** traumatic brain injury, pediatric TBI, neuroinflammation, biomarkers, neuroimaging, brain injury

## Abstract

Traumatic Brain Injury (TBI) in children is a profound public health issue with the potential to disrupt cognitive, behavioral, and psychosocial development significantly. This review provides an updated examination of the role of neuroinflammation in pediatric TBI, emphasizing its dual impact on injury progression and recovery. Highlighted is the complex interplay of primary and secondary injury mechanisms, including the critical contributions of neuroinflammatory responses mediated by central and peripheral immune cells. Advances in biomarker identification and imaging techniques are discussed, showcasing how tools like diffusion tensor imaging (DTI) and positron emission tomography (PET) enhance our understanding of neuroinflammatory processes. The review also explores current therapeutic strategies targeting neuroinflammation, underscoring emerging treatments such as pharmacologic agents that modulate immune responses and novel therapies like stem cell interventions. This comprehensive review seeks to deepen the understanding of neuroinflammation’s pathophysiological roles in pediatric TBI and propose directions for future clinical and research efforts.

## Introduction

Traumatic brain injury (TBI) remains a critical public health challenge worldwide, particularly as one of the leading causes of disability and mortality among children and adolescents (Jacquens et al., 2024; Lajud et al., 2021; Fraunberger and Esser, 2019). In 2022, the US Centers for Disease Control and Prevention (CDC) reported that 2.3 million children <18 years old had ever received a diagnosis of concussion or brain injury (Elgaddal and Black, 2023). Additionally, a 2017 analysis using data from the CDC’s *Mortality and Morbidity Weekly Report* (MMWR) reported that the incidence of TBI-related emergency department (ED) visits, hospitalizations, and mortality within the pediatric population was roughly 1% (Taylor et al., 2017). The long-term consequences of TBI in this demographic can severely disrupt cognitive, behavioral, and psychosocial development. While the impacts of pediatric TBI on areas such as executive function and cognitive flexibility are well-documented, the broader literature also extensively discusses the myriad causes of TBI in children, from falls and motor vehicle collisions to sports injuries and child abuse. Each cause presents distinct challenges in clinical management and prognosis (Jacquens et al., 2024; Lajud et al., 2021; Fraunberger and Esser, 2019; Gao et al., 2020; Hon et al., 2019; Emery et al., 2016; Peterson et al., 2019). Additionally, the classification of TBI severity in children has often been extrapolated from the adult literature and is based on the Glasgow Coma Scale (GCS) which is scored from 3 to 15: mild TBI (13–15), moderate TBI (9–12), severe TBI (3–8) (Haydel et al., 2025; Jain and Iverson, 2025).

Substantial research has been conducted on the immediate and secondary injury phases in pediatric TBI, which are characterized by mechanical damage and a complex biochemical cascade, respectively (Jacquens et al., 2024; Lajud et al., 2021; Fraunberger and Esser, 2019; Gao et al., 2020; Kölliker-Frers et al., 2021; Reis et al., 2017; Regner et al., 2018; Sulhan et al., 2020; Xiong et al., 2018; Burda et al., 2016; Pearn et al., 2017; Wolf et al., 2001; Zetterberg et al., 2013; De Souza et al., 2009; Roth et al., 2014; Liddelow et al., 2017; Tang-Schomer et al., 2010; Winkler et al., 2016; Chodobski et al., 2011; Abdelhak et al., 2022; Yue et al., 2019; Pelinka et al., 2004; Sperry et al., 2024; Okonkwo et al., 2020; Thelin et al., 2017; Oris et al., 2023; Park and Hwang, 2018; Cheng et al., 2014; Bandyopadhyay et al., 2005; Wilkinson et al., 2017; Czeiter et al., 2020; Agoston et al., 2017; Rosenberg et al., 1997; Zou et al., 2021; Park, 2018; Xie et al., 2024; Gonzalez Rodriguez et al., 2018; Iczkiewicz et al., 2007). It is evident that neuroinflammation plays a critical dual role. Initially beneficial for debris clearance and neuronal repair, prolonged or dysregulated inflammation can impair recovery and exacerbate long-term outcomes (Jacquens et al., 2024; Hamood et al., 2022). Recent studies have proposed that such sustained inflammation could lead to glymphatic dysfunction, which impedes the brain’s waste-clearance mechanisms, thereby accumulating toxic metabolites and potentially accelerating neurodegeneration (Peters and Lyketsos, 2023). Amid ongoing brain development and heightened neuroplasticity in children, understanding the nuanced responses to brain injury is crucial for improving long-term outcomes. This review acknowledges the robust discussion in existing literature surrounding key inflammatory mediators like cytokines and chemokines (e.g., TNF-*α*, IL-1β, IL-6, HMGB1), which are central both in the pathology and the resolution of TBI (Abdelhak et al., 2022; Yue et al., 2019; Pelinka et al., 2004; Sperry et al., 2024; Okonkwo et al., 2020; Thelin et al., 2017; Oris et al., 2023; Park and Hwang, 2018; Cheng et al., 2014; Bandyopadhyay et al., 2005; Wilkinson et al., 2017; Czeiter et al., 2020; Agoston et al., 2017; Zou et al., 2021; Park, 2018; Xie et al., 2024; Gonzalez Rodriguez et al., 2018; Iczkiewicz et al., 2007; Rogan et al., 2023; Undén and Romner, 2010; Ergün et al., 1998; Chen et al., 2022). Furthermore, recent emphasis on identifying reliable biomarkers such as plasma osteopontin and glial fibrillary acidic protein highlights advances in diagnosing and predicting the progression of pediatric TBI (Jacquens et al., 2024; Gao et al., 2020; Abdelhak et al., 2022; Yue et al., 2019; Pelinka et al., 2004; Sperry et al., 2024; Iczkiewicz et al., 2007).

This manuscript integrates recent findings to deepen our understanding of neuroinflammation’s pathophysiological roles in pediatric TBI. It strives to expand on the current literature by offering new insights into the inflammatory mechanisms, examining both systemic and localized responses and their effects on neurological health over time. By proposing future directions for clinical and research efforts, this review seeks to fill gaps in our understanding and suggest ways to mitigate the long-term impacts of pediatric TBI.

## Pathophysiology of neuroinflammation in pediatric TBI

The inflammatory response to traumatic brain injury (TBI) involves a complex interplay between various central nervous system (CNS) and peripheral immune cells, each contributing to the overall inflammatory cascade. Astrocytes, microglia, pericytes, and mast cells play crucial roles in the CNS response, while neutrophils, monocytes, and T cells drive the peripheral response (Harting et al., 2008). Understanding these mechanisms and their interactions is essential for developing targeted therapies for TBI. While the complexity of outcomes following CNS injuries in the developed brain is extensive, injuries to the developing brain pose a unique and significant challenge. The ongoing processes of synaptogenesis and myelination in the developing brain are crucial for forming neural networks that may be disrupted by an injury that is mild in severity, which can impact behavior and molecular interactions, such as inflammation. One of the significant consequences of TBI-induced neuroinflammation is the subsequent activation of microglia, the brain’s resident immune cells, which serve a multifaceted role in both the injury response and downstream recovery (Donat et al., 2017). While microglia are essential for removing debris and modulating repair mechanisms, overactivation leads to increased synaptic pruning, disrupting normal synaptogenesis and myelination. This dysregulated microglial activity impairs the stability of neural circuits, particularly in white matter structures, contributing to long-term cognitive and motor deficits (Eyolfson et al., 2020).

Traumatic brain injury (TBI) is a leading cause of death and disability across all age groups worldwide (Hon et al., 2019). Due to the ongoing brain development in children, this group is particularly susceptible to both acute and chronic cognitive and behavioral changes following a TBI. These changes include sleep disturbances, impaired attention and memory, emotional instability, and motor and balance issues (Emery et al., 2016). TBI is defined by the Centers for Disease Control (CDC) as a disruption in brain function caused by an external force to the head, otherwise referred to as the “primary” injury (Peterson et al., 2019). The injury-inducing forces can be applied in a linear or non-linear (i.e., rotational) fashion, which may physically damage brain tissue. The pathophysiology of pediatric TBI involves a multifaceted interplay of primary and secondary injury mechanisms.

The primary injury in TBI occurs immediately following the impact that results in physical disruption of brain tissue, including neurons, microglia, and endothelial cells that makeup the blood–brain barrier (BBB) (Reis et al., 2017). The effects following the initial insult trigger a cascade of secondary injury processes characterized by ischemia, hypoxia, cerebral edema, raised intracranial pressure, excitotoxicity, calcium dysregulation, mitochondrial dysfunction, inflammation, and apoptosis (Regner et al., 2018; Sulhan et al., 2020; Xiong et al., 2018). These secondary injury mechanisms evolve over a longer time span, ranging from hours, days, and weeks, following the initial trauma, which exacerbates the primary injury, leading to further neurological damage (Hamood et al., 2022).

**Figure 1:**
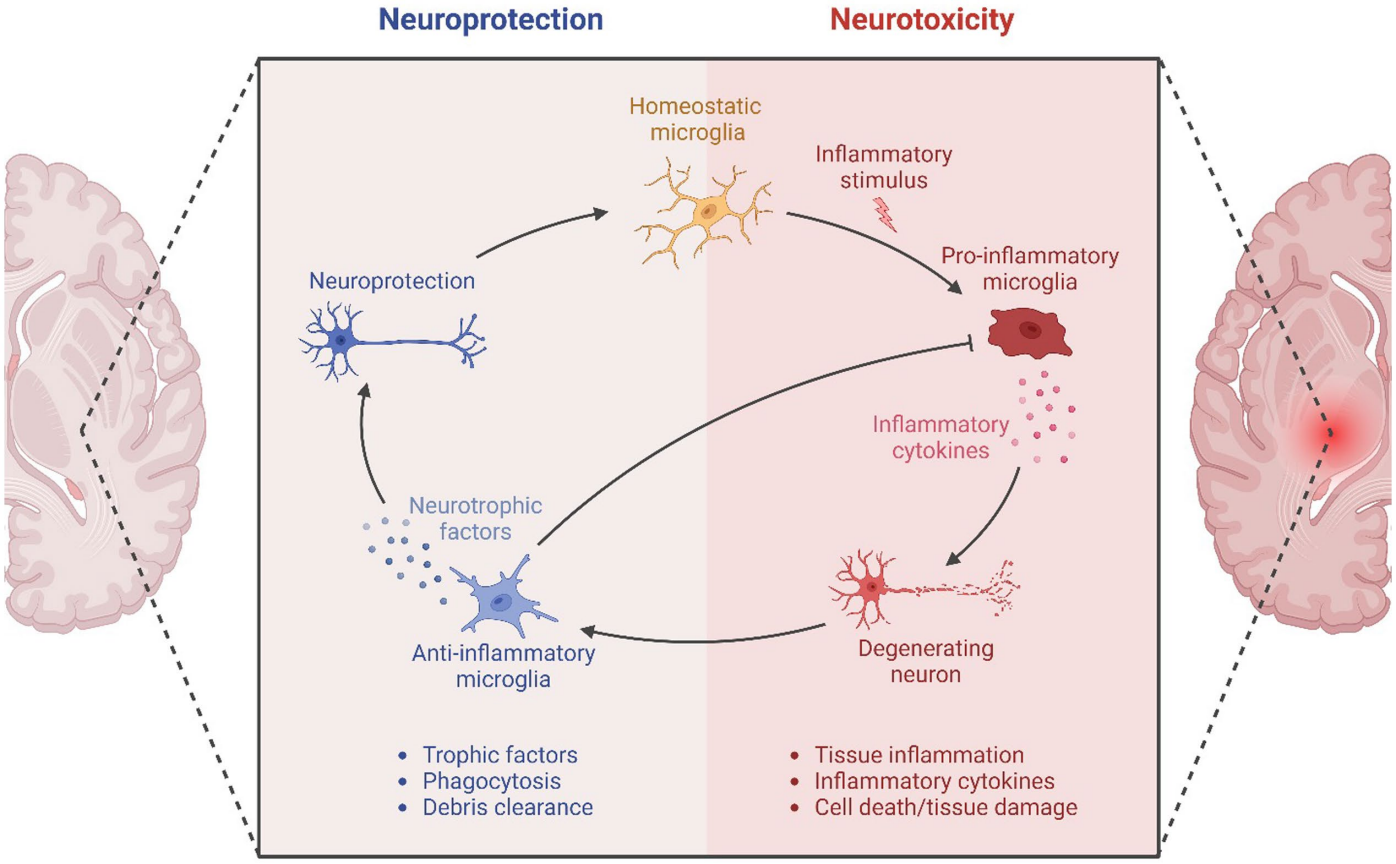
Shows the process of neuroinflammation and the role of the immune system in promoting neuroprotection and neurotoxicity (https://biorender.com/e81a861). Modified from https://app.biorender.com/biorender-templates/figures/all/t-63a4bc394320eed93210b91b-roles-of-microglia-in-neuroinflammation.

The secondary cascade involved in pediatric TBI involves the activation of a neuroinflammatory response involving microglia, astrocytes, and peripheral immune cells, which disrupts the integrity of the BBB. TBI-induced activation of astrocytes results in the stretching of the N-methyl-D-aspartate (NMDA) surface receptors, causing deformity and unregulated ionic changes (Burda et al., 2016). The range of severity in the primary injury determines the degree of cellular membrane damage, which results in the rapid and unregulated influx of sodium coupled with potassium efflux and increases in intra-axonal calcium (Pearn et al., 2017; Wolf et al., 2001; Zetterberg et al., 2013). Simultaneous ATP release initiates autocrine and paracrine calcium influx, causing additional ATP, glutamate, and S100B release through MAPK signaling (Jacquens et al., 2024; Pearn et al., 2017). This process leads to the activation of astrocytes and microglia recruitment to the site of injury, which secrete IL-1a, TNFa, and C1q, stimulating a phenotypic conversion of astrocytes to an A1 “neurotoxic” profile, demonstrating the reciprocal activation of these glial cells in TBI (Roth et al., 2014; Liddelow et al., 2017). In addition to the ionic mechanism changes, rapid acceleration and deceleration upon impact results in extensive shearing of axons, known as diffuse axonal injury. Structural analysis of axons following injury shows microtubule breakage, eventually leading to the progressive disassembly of microtubules (Tang-Schomer et al., 2010). The damage to these structures contributes to the accumulation of protein products, interruption of axonal transport, axonal swelling, and degeneration (Tang-Schomer et al., 2010). Figure 1 illustrates the role of microglia and the immune system in promoting neurotoxicity.

Peripheral immune cells play a pivotal role in the inflammatory response following TBI in the pediatric population. Previous studies have indicated the differences in peripheral immune cell composition in adolescents compared to adults for up to 18 years. These differences include, for example, lymphocytes slowly increasing in children from ages 6 months to 18 years old, while neutrophils and eosinophils decrease in ages 14–18 (Aldrimer et al., 2013). This is an important indication as the cells responding to the site of injury will differ between the pediatric and adult populations, greatly altering the immune response and subsequent inflammation. The disruption of BBB permeability following TBI leads to the recruitment of macrophages, neutrophils, and lymphocytes to the injury site, further contributing to the inflammatory response. Injured brain parenchyma increases the expression of leukocyte adhesion molecules on the brain endothelium early in TBI (Worthylake and Burridge, 2001). Through this cascade, leukocytes further disrupt the BBB by generating reactive oxygen species, activating proteolytic enzymes, and secreting cytokines and chemokines (Winkler et al., 2016; Chodobski et al., 2011). Additionally, the chemokines secreted attract more inflammatory cells, allowing for their extravasation across the vascular membrane, furthering the neuroinflammatory response and damage to brain tissue. Understanding the pathophysiology of pediatric TBI requires consideration of both the unique aspects of the developing brain and the complex interplay of inflammatory and immune responses. Further research is needed to develop effective treatments that address these intricate mechanisms.

## Biomarkers and neuroinflammation

In the evolving landscape of traumatic brain injury (TBI) research, molecular biomarkers are paramount in advancing our understanding of the complex pathology that characterizes TBI. Traditional biomarkers such as Glial fibrillary acidic protein (GFAP), S100B, and neuron-specific enolase (NSE) are well-documented in their roles in parenchymal trauma. GFAP, a protein predominantly found in astrocytes, is utilized to evaluate astrocytic injury and has been correlated with injury severity and patient outcomes, often serving as a reliable indicator of the structural impact of TBI (Abdelhak et al., 2022; Yue et al., 2019; Pelinka et al., 2004; Sperry et al., 2024; Okonkwo et al., 2020). S100B serves a dual role, primarily reflecting acute neuronal damage due to its presence in mature perivascular astrocytes and its involvement in calcium signaling and regulation. It has been effectively used in clinical settings to predict neurological outcomes following TBI (Thelin et al., 2017; Oris et al., 2023; Park and Hwang, 2018; Rogan et al., 2023; Undén and Romner, 2010). NSE, highly specific to neurons, is another critical marker for assessing the extent of neuronal damage and is often utilized to gauge the severity of brain injuries and to predict long-term recovery trajectories (Cheng et al., 2014; Bandyopadhyay et al., 2005; Wilkinson et al., 2017; Ergün et al., 1998; Chen et al., 2022). In addition to these markers of primary injury, or focal biomarkers, UCHL-1, total tau, NF-L and MBP are also being studied as potential focal biomarkers (Sperry et al., 2024; Czeiter et al., 2020; Agoston et al., 2017).

While focal biomarkers offer valuable insights into the extent of structural damage, they fall short in providing direct measures of the neuroinflammatory processes that play a critical role in the secondary injury mechanisms following TBI. This gap has led to increased research into biomarkers that can specifically quantify neuroinflammation, or global biomarkers.

Among the emerging global biomarkers, Osteopontin (OPN) and Syndecan-1 (sdc1) have shown promise in specifically reflecting the inflammatory response in TBI. OPN is involved in regulating immune processes and is upregulated in response to trauma, particularly in microglial cells. Elevated levels of OPN not only correlate with the severity of the injury but also with inflammatory processes that may affect recovery and long-term outcomes (Gao et al., 2020). This biomarker offers a unique perspective on the inflammatory landscape following TBI, providing insights that are not captured by traditional biomarkers focused on structural damage. Syndecan-1, a component of the endothelial extracellular matrix, is crucial for vascular integrity and is implicated in the systemic inflammatory response following TBI. Studies have shown that elevated levels of sdc1 after TBI indicate endothelial damage and a broader inflammatory response that may worsen neurological outcomes, particularly in patients with concurrent extracranial injuries (Rosenberg et al., 1997; Zou et al., 2021; Park, 2018; Xie et al., 2024; Gonzalez Rodriguez et al., 2018). The measurement of sdc1 levels provides a window into the systemic effects of TBI and highlights the interconnectedness of inflammatory pathways that extend beyond the primary site of injury.

Furthermore, the exploration of biomarkers like High Mobility Group Box 1 (HMGB1) and Interleukin-6 (IL-6) is expanding our understanding of the molecular dynamics at play in TBI. HMGB1, a nuclear protein released during cellular stress and necrosis, acts as a pro-inflammatory mediator in the brain, exacerbating tissue damage following TBI. Its role as a biomarker is significant in delineating the mechanisms of secondary injury and inflammation (Iczkiewicz et al., 2007; Wozniak et al., 2007; Dennis et al., 2018; Bodanapally et al., 2019; Bodanapally et al., 2019). Similarly, IL-6 is rapidly upregulated following TBI and has been studied for its dual role as both a pro-inflammatory cytokine and a potential marker for therapeutic targets, especially in modulating the acute inflammatory response to improve patient outcomes.

While considering the potential for clinical utility of biomarkers in the context of TBI, it is important that their limitations be given due consideration. NSE, which is also found in erythrocytes, loses specificity in settings where hemolysis can significantly confound its interpretation. Although S100B is immune to this interference, its short half-life makes its sensitivity heavily dependent on the delay between initial injury and its measurement (Oris et al., 2023). Sdc1, given its global expression in vasculature throughout the body, loses specificity in polytrauma settings when extracranial injuries can contribute to elevations in its levels (Xie et al., 2024).

Though the utility and significance of various biomarkers in TBI is an area of active research, there have already been promising clinical applications. Of particular interest are GFAP and UCHL-1, markers of glial and neuronal injury, respectively. These biomarkers, in tandem, have demonstrated significant diagnostic and prognostic value for the management of intracranial injury (Bazarian et al., 2018; Posti et al., 2016). Furthermore, a rapid blood test to assay them has recently gained FDA approval, facilitating their transition from the bench to the bedside. In the pediatric population, this tool was demonstrated to have a sensitivity and negative predictive value of 100% for the detection of clinically important traumatic brain injuries (Puravet et al., 2025). Though significant, this work alludes to the greater promise that biomarkers hold for guiding clinical management and prognostication. Further work directed toward the integration of these biomarkers into clinical practice is crucial for developing targeted therapies that address both the immediate and secondary injuries associated with TBI. By better understanding the specific roles of these biomarkers, particularly those involved in neuroinflammation, clinicians and researchers can tailor treatments that not only mitigate the initial impact of TBI but also the prolonged inflammatory responses that can dictate patient recovery and quality of life.

## Imaging techniques

Advancements in imaging techniques have significantly improved our understanding of neuro-inflammatory changes following pediatric traumatic brain injury (TBI). While MRI, including diffusion tensor imaging (DTI), has been widely recognized for its sensitivity to white matter integrity and axonal damage, other advanced technologies such as dual-energy computed tomography (DECT) also play a crucial role in neuro-inflammation research.

DTI is invaluable for its ability to measure the diffusion properties of water molecules within brain tissue, providing detailed insights into white matter disruptions indicative of neuro-inflammatory responses. This MRI technique uses parameters such as fractional anisotropy (FA) and mean diffusivity (MD) to offer a nuanced view of the microstructural changes that are characteristic of inflammation following TBI. DTI’s sensitivity to axonal integrity makes it an excellent tool for detecting diffuse axonal injury (DAI), a common feature in TBI that is closely associated with inflammatory processes (Wozniak et al., 2007; Dennis et al., 2018; Bodanapally et al., 2019; Bodanapally et al., 2019; Basser and Jones, 2002; Basser et al., 1994; Wallace et al., 2018; Arfanakis et al., 2002; Hulkower et al., 2013; Huisman et al., 2004; Roberts et al., 2016; Roberts et al., 2014; Wilde et al., 2008).

DECT, as an advanced imaging technology, extends beyond traditional applications by offering unique capabilities in assessing material composition and structural integrity within the brain. This technique is particularly useful for detecting variations in brain tissue that may signify inflammation, such as the presence of hemorrhagic contusions, which are often associated with traumatic brain injuries. DECT’s ability to image at two different energy levels allows for superior differentiation of tissue types and provides a clearer depiction of the brain’s condition post-injury (Gibney et al., 2020; Hamid et al., 2021; Hu et al., 2016; Tanzil et al., 2023; Bodanapally et al., 2019; Lee et al., 2023).

Furthermore, the use of positron emission tomography (PET) with probes such as the mitochondrial translocator protein 18-kDa (TSPO) enriches our capability to visualize and quantify neuro-inflammation directly. TSPO, released by activated microglia and astrocytes, is a critical marker in neuro-inflammatory studies. Research continues to enhance the specificity of TSPO PET tracers and refine imaging protocols, aiming to provide more precise diagnostic and therapeutic outcomes (Chen et al., 2021; Zhang et al., 2021; Schubert et al., 2021; Wimberley et al., 2021; Nikam et al., 2021). Tau deposits are also a core pathological feature of TBI (Wang et al., 2018; Sarkis et al., 2021). Tau PET imaging with tracers such as 11C-PBB3 has demonstrated that increased tracer binding in neocortical and white matter regions in long-term TBI survivors correlates with late-onset neuropsychiatric symptoms, including psychosis (Takahata et al., 2019). Notably, patients with traumatic encephalopathy syndrome show significantly higher 11C-PBB3 binding in the frontal white matter, and *in vitro* assays confirm that this binding reflects tau lesions, particularly at the depths of neocortical sulci. The development of new PET probes targeting other elements of the inflammatory pathway such as cyclooxygenase-1 (COX-1), cyclooxygenase-2 (COX-2), and cannabinoid type 2 (CB2) receptors is also promising, potentially offering deeper insights into the complex inflammatory responses in pediatric TBI (Kim et al., 2020; Prabhakaran et al., 2021; Haider et al., 2019; Bedi et al., 2023).

The integration of DTI, DECT, and PET into the diagnostic process not only enriches our understanding of the structural damages associated with TBI but also illuminates the extensive neuro-inflammatory reactions that are critical to developing effective treatment strategies.

## Therapeutic options

In addressing the treatment of pediatric traumatic brain injury (TBI), a focused discussion on interventions targeting neuro-inflammation is critical given its pivotal role in both the progression and outcome of TBI. Pharmacologic treatments such as Minocycline and Intravenous Immunoglobulin (IVIG) modulate immune responses and reduce microglial activation, crucial in the secondary injury cascade. These agents have shown promise in mitigating neurodegeneration following acute neuro-inflammatory responses, although further research is needed to fully ascertain their efficacy (Liu et al., 2023; Koulaeinejad et al., 2019; Thom et al., 2017). Antioxidants like N-acetylcysteine, vitamin C, and vitamin E combat oxidative stress—an aftermath of increased reactive oxygen species and lipid peroxidation post-TBI, helping to protect against further cellular injury and support recovery from neuro-inflammatory damage (Davis and Vemuganti, 2022).

Emerging therapies, including stem cell treatments, represent a novel frontier in TBI management. Research on neural stem cells (NSCs) and mesenchymal stem cells (MSCs) has shown potential in regenerating damaged neural tissue and aiding recovery from neurodegenerative impacts of sustained neuro-inflammation (Schepici et al., 2020; Weston and Sun, 2018; Walker et al., 2012; Walker et al., 2011; Savitz and Cox, 2023; Cox et al., 2024; Caplan et al., 2021). These therapies, however, remain largely experimental and require more rigorous trials to establish their safety and effectiveness in pediatric populations.

Surgical interventions such as decompressive craniectomy are essential for managing severe cases where pharmacologic and non-invasive methods are insufficient. Primary decompressive craniectomy is performed urgently to mitigate acute brain swelling and prevent herniation in cases where there is significant brain compression or a deteriorating neurological examination due to an expanding hematoma (Sy et al., 2021; Trimble et al., 2020). Secondary decompressive craniectomy, on the other hand, is considered for late refractory intracranial pressure (ICP) elevation that is unresponsive to other treatments. This surgical approach is only recommended when ICP remains dangerously high despite exhaustive medical interventions, providing a necessary relief to preserve brain function and manage inflammation effectively (Hawryluk et al., 2020). These procedures, particularly when paired with less invasive interventions such as ICP monitoring and CSF drainage, form a comprehensive treatment strategy that addresses both the immediate and secondary impacts of neuro-inflammation in pediatric TBI. By integrating a range of therapeutic approaches—from medical management to surgical intervention—treatment plans can be tailored to effectively manage the complex challenges posed by severe TBI, ultimately improving outcomes and prognoses for affected children. However, it is important to note, that only a small fraction of pediatric TBI is severe enough to necessitate surgical intervention (Dewan et al., 2016).

A vast majority of pediatric TBI is classified as mild or moderate with negative imaging (Dewan et al., 2016; Greenberg et al., 2019; Venkataraman et al., 2018). Management of these children requires a multifaceted approach, encompassing both medical and rehabilitative strategies to optimize recovery and prevent long-term complications. The initial phase of management focuses on accurate diagnosis and symptom monitoring, as persistent post-concussive symptoms can impact cognitive, emotional, and physical functioning (Lumba Brown et al., 2018). Early intervention with cognitive and physical rest is essential, but recent guidelines emphasize a gradual return to activity rather than prolonged restriction, as excessive inactivity may exacerbate symptoms and prolong recovery (Levin and Diaz-Arrastia, 2015; Kirkwood et al., 2008). Non-pharmacological therapies, including cognitive rehabilitation, speech therapy, occupational therapy, and physical therapy, are crucial for managing deficits in attention, memory, balance, and motor function, especially in cases where symptoms persist beyond the acute phase (Kirkwood et al., 2008). Additionally, emotional and psychological support is necessary, as mTBI can contribute to mood disorders, anxiety, and behavioral changes (Lumba Brown et al., 2018). Parental education plays a key role in reinforcing symptom monitoring and ensuring adherence to activity restrictions and rehabilitation plans (Levin and Diaz-Arrastia, 2015). While most children recover within weeks to months, a subset may experience prolonged symptoms requiring specialized interventions, including neuropsychological assessment and targeted therapy (Lumba Brown et al., 2018; Kirkwood et al., 2008).

Overall, these approaches highlight the importance of individualized and multifaceted strategies that incorporate both innovative and traditional treatments to combat the diverse manifestations of TBI in the pediatric population and improve long-term functional outcomes.

## Conclusion

This review has articulated the critical roles neuroinflammation plays in the pathophysiology of pediatric traumatic brain injury (TBI), delineating its profound impact on long-term outcomes. We have explored the nuanced interactions between mechanical injury and subsequent biochemical cascades, emphasizing how these interactions lead to persistent inflammatory responses that can drastically alter neurological development and recovery. Advancements in biomarker technology and neuroimaging have been pivotal in enhancing our understanding of these complex processes. The identification of specific biomarkers like OPN and sdc and emerging PET probes tailored to neuroinflammatory markers offers promising avenues for more precise diagnosis and monitoring of TBI progression. Furthermore, imaging modalities such as DTI and PET have provided unparalleled insights into the microstructural and metabolic changes associated with TBI, allowing for a more targeted approach in clinical assessments and interventions.

Despite these advancements, the treatment of pediatric TBI remains challenging, with current therapeutic strategies primarily focusing on mitigating immediate risks and stabilizing the patient. The exploration of new treatments, particularly those targeting the inflammatory and degenerative components of TBI, such as the use of minocycline, IVIG, and stem cell therapies, opens new doors for potentially transformative outcomes. However, the efficacy of these novel interventions requires robust validation through clinical trials to ensure they are both safe and effective for pediatric use. As we move forward, it is imperative that the research community continues to develop and refine strategies that address both the acute and chronic aspects of neuroinflammation in pediatric TBI. Collaborative efforts, including multi-center trials and longitudinal studies, will be essential in overcoming the current limitations in treatment and improving the prognosis for affected children. Ultimately, by deepening our understanding of neuroinflammation’s mechanisms and consequences, we can pave the way for innovative therapeutic approaches that significantly enhance recovery and quality of life for young TBI patients.
